# Research progress on chemical composition, pharmacological effects, clinical applications, and processing of *Curcumae Radix*

**DOI:** 10.3389/fphar.2026.1875758

**Published:** 2026-07-09

**Authors:** Linlin Wang, Qiao Zhou, Juanjuan Zhu, Qi Lu, Lei Zhang, Meng Jin, Yanpeng Dai, Dianhua Shi

**Affiliations:** 1 Institute of Traditional Chinese Medicine Processing, Shandong Academy of Chinese Medicine, Jinan, Shandong, China; 2 Institute of Brain Science and Brain-inspired Research, Shandong Provincial Key Laboratory of Brain Science, Shandong First Medical University & Shandong Academy of Medical Sciences, Jinan, Shandong, China

**Keywords:** chemical composition, clinical application, curcumae radix, pharmacology, processing

## Abstract

*Curcumae Radix*: the dried root tuber of plants from the genus *Curcuma* (family *Zingiberaceae*), is a commonly used Chinese materia medica for promoting blood circulation and moving qi. It exhibits effects including invigorating blood circulation to relieve pain, moving qi to resolve depression, clearing the heart to cool blood, and promoting bile flow to relieve jaundice. Its botanical origins include *Curcuma wenyujin*, *Curcuma longa*, *Curcuma kwangsiensis*, and *Curcumae phaeocaulis*. Modern investigations have shown that *Curcumae Radix* contains over 250 compounds, mainly volatile oils and curcuminoids. Pharmacologically, it exhibits antitumor, hepatoprotective, anti-inflammatory, analgesic, cardiovascular-protective, and neuroprotective effects. In clinical practice, it is often combined with other herbs for the synergistic treatment of hepatobiliary, neurological, and digestive system disorders. Processing can alter the dissolution and transformation of its chemical constituents, thereby enhancing its efficacy or directionally regulating its actions. Although several reviews have summarized the chemical composition and pharmacological effects of *Curcumae Radix*, there is still a lack of clear understanding regarding the correlation between chemical differences and efficacy among different botanical origins, a systematic elucidation of the molecular mechanisms underlying processing-induced efficacy enhancement, and high-quality clinical translational evidence. This review systematically summarizes the chemical constituents, pharmacological effects, clinical applications, and processing research progress of *Curcumae Radix*, identifies the current key knowledge gaps, and provides a reference for the precision clinical application and modern development of this herbal medicine.

## 1 Introduction

*Curcumae Radix*, the dried root tuber of plants belonging to the genus *Curcuma* (family *Zingiberaceae*), is a typical multi-origin Chinese materia medica. Its core efficacies, recorded in herbal classics through the ages, include activating blood circulation and alleviating pain, moving qi and relieving depression, clearing heart and cooling blood, promoting bile flow and relieving jaundice, which have been applied to the present day (Ao et al., 2022; Committee, 2025).

Modern studies have isolated and identified over 250 compounds from *Curcumae Radix*, among which volatile oils, curcuminoids, and polysaccharides are the core pharmacodynamic substances. Its pharmacological effects cover antitumor, hepatoprotective, anti-inflammatory and analgesic, antithrombotic, and neuroprotective activities (Niu et al., 2024). *Curcumae Radix* has shown unique clinical value in hepatobiliary, cardiovascular, neuropsychiatric, and dermatological diseases (Chen et al., 2023; Li, 2022). The processing of *Curcumae Radix* began in the Tang Dynasty, with vinegar processing and wine processing being the mainstream modern methods, which can significantly alter the composition and direction of pharmacological effects (Chen et al., 2018).

Although several reviews have summarized the chemical composition and pharmacological effects of *Curcumae Radix*, notable research gaps remain. First, the four officially recognized botanical origins exhibit significant chemical differences; however, the correlation between these chemical variations and their pharmacological efficacy remains unclear, and the different origins are often used interchangeably in clinical practice. Second, the molecular mechanisms underlying the efficacy enhancement or directional regulation of pharmacological actions induced by processing methods (e.g., vinegar-processing and wine-processing) lack systematic elucidation. Third, high-quality clinical translational evidence is still scarce, which hinders the precision medicine application and modern development of *Curcumae Radix*.

This review attempts to establish an integrated framework linking botanical origin, chemical composition, pharmacological activity, clinical application, and processing-induced modulation. Specifically, we distinguish three levels of evidence: direct comparative evidence among different botanical origins, indirect evidence inferred from characteristic constituents and their known bioactivities, and clinical evidence derived from traditional prescriptions or modern clinical observations. This framework allows a more critical evaluation of the current literature and highlights the key gaps that must be addressed for the precision use of *Curcumae Radix*, aiming to provide ideas for its in-depth development and rational clinical application.

## 2 Medicinal plant origin

*Curcumae Radix* is a typical multi-origin Chinese medicinal material, namely, *C. wenyujin, C. longa, C. kwangsiensis, and C. phaeocaulis*. According to their morphological characteristics and producing areas, they are commonly known as *Wenyujin*, *Huangsiyujin*, *Guiyujin*, and *Lvsiyujin*, respectively.

The genus *Curcuma* is the third largest genus in the Zingiberaceae family, comprising approximately 80 species worldwide, mainly distributed in Southeast Asia. More than 10 species are distributed in China, concentrated in the southwestern to southeastern regions. All four botanical origins are perennial herbs, characterized by fleshy and aromatic rhizomes and swollen terminal root tubers. They bear large basal leaves, produce a spike-shaped inflorescence containing mucus, and have funnel-shaped corollas (Flora, 2019). The morphological characteristics and geographical distributions of the four origins are summarized in Table 1.

**TABLE 1:** Morphological and distribution characteristics of the four botanical origins of *Curcumae Radix*.

Botanical origin	Plant height	Leaf characteristics	Root characteristics	Flowering period	Main distribution
*C. wenyujin*	∼1 m	Root tubers fusiform, swollen at the end	Apex with fine tail-like tip, base gradually narrowed, glabrous	April–June	Zhejiang
*C. longa*	1–1.5 m	Roots robust, swollen at the end into tuberous roots	Apex shortly acuminate, base gradually narrowed, both surfaces glabrous	August	Taiwan, Fujian, Guangdong, Guangxi, Yunnan
*C.kwangsiensis*	∼1 m	Roots slender, growing around the rhizome, often swollen into nearly fusiform tubers at the end	Apex shortly acuminate to acuminate, margins slightly revolute, both surfaces pubescent	May–July	Guangxi, Yunnan
*C.phaeocaulis*	∼1 m	Roots slender or swollen at the end into tuberous roots	Erect, often with purple spots in the center, glabrous	April–June	Guangxi, Yunnan, Sichuan

## 3 Chemical composition

The chemical composition of *Curcumae Radix* is complex and diverse, primarily containing two major groups of active components: volatile oils and diarylheptanoids (also known as curcuminoids), along with various other constituents such as polysaccharides, alkaloids, sterols, flavonoids, and trace elements. To date, over 250 compounds have been isolated and identified from *Curcumae Radix*, among which sesquiterpenoids are the most abundant (161 compounds), followed by monoterpenes (27 compounds) and diarylheptanoids (19 compounds) (Niu et al., 2024).

### 3.1 Volatile oil components

Volatile oils are the main active fraction of *Curcumae Radix*, predominantly composed of terpenoids, among which sesquiterpenoids are the key components and serve as an important material basis for its pharmacological effects, including antitumor, anti-inflammatory, antithrombotic, and antiviral activities. The volatile oil content varies significantly among different botanical origins of *Curcumae Radix*, with *C*. *longa* exhibiting the highest content (1.2%–2.0%), while the other varieties range from 0.1% to 0.7% (Ao et al., 2022).

**FIGURE 1:**
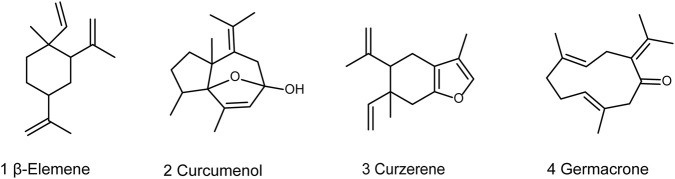
Chemical structures of representative volatile oils components in *Curcumae Radix*. (1) β-Elemene (elemane type), (2) Curcumenol (guaiane type), (3) Curzerene (guaiane type), (4) Germacrone (germacrane type).

Based on differences in the skeletal structure, the sesquiterpenes in *Curcumae Radix* can be classified into several types, including guaiane, germacrane, bisabolane, eudesmane, and elemane types (Li H. L. et al., 2022). Representative compounds include curcumol (guaiane type), germacrone (germacrane type), and β-elemene (elemane type) (Ao et al., 2022; Chen et al., 2025a; Chen et al., 2025b; Li Y. et al., 2022; Shen et al., 2014). Monoterpenes including limonene, camphor, and linalool have also been identified (Liu, 2007). Representative volatile oil components are listed in Table 2 and Figure 1.

**TABLE 2:** Representative volatile oil components in Curcumae Radix.

Classification	Chemical constituent	Molecular formula	Botanical origin	Ref
Guaiane type	Curcumol	C_15_H_24_O_2_	*C. wenyujin, C. kwangsiensis*	Lan (2024)
	Curcumenol	C_15_H_22_O_2_	*C. wenyujin, C. phaeocaulis*	Lan (2024)
	Isocurcumenol	C_15_H_22_O_2_	*C. wenyujin, C. kwangsiensis, C. phaeocaulis*	Lan (2024)
Germacrane type	Curdione	C_15_H_24_O_2_	*C. wenyujin, C. kwangsiensis, C. phaeocaulis*	Hu et al. (2016), Li Y. et al. (2022), Song et al. (2010)
	Germacrone	C_15_H_22_O	*C. wenyujin, C. kwangsiensis, C. phaeocaulis*	Lan et al. (2024)
	Furanodiene	C_15_H_20_	*C. wenyujin, C. kwangsiensis, C. phaeocaulis*	Lan et al. (2024)
Bisabolane type	ar-Curcumene	C_15_H_22_	*C. longa, C. wenyujin*	Li Y. et al. (2022)
	ar-Turmerone	C_15_H_20_O	*C. longa*	Zeng et al. (2007)
Eudesmane type	β-Eudesmol	C_15_H_26_O	*C. wenyujin, C. kwangsiensis*	Cai et al. (2018)
Elemane type	β-Elemene	C_15_H_24_	*C. wenyujin, C. kwangsiensis*	Lan et al. (2024)
	γ-Elemene	C_15_H_24_	*C. wenyujin, C. kwangsiensis*	Wang Y. et al. (2024)
	δ-Elemene	C_15_H_24_	*C. wenyujin, C. kwangsiensis*	Wang J. A. et al. (2024)
	Curzerenone	C_15_H_18_O	*C. wenyujin*	Zhou Y. et al. (2017)
	Curzerene	C_15_H_18_	*C. wenyujin*	Lan et al. (2024)
p-Menthane type	Limonene	C_10_H_16_	*C. wenyujin, C. longa, C. kwangsiensis*	Cai et al. (2018)
	Terpinen-4-ol	C_10_H_18_O	*C. wenyujin, C. longa*	Cai et al. (2018)
	α-Terpinene	C_10_H_18_O	*C. wenyujin, C. longa*	Cai et al. (2018)
Bornane type	Camphor	C_10_H_16_O	*C. wenyujin, C. kwangsiensis*	Zhang et al. (2024)
	Borneol	C_10_H_18_O	*C. wenyujin, C. kwangsiensis*	Zhang et al. (2024)
Pinane type Others	α-Pinene	C_10_H_16_	*C. wenyujin, C. longa, C. kwangsiensis*	Cai et al. (2018)
	β-Pinene	C_10_H_16_	*C. wenyujin, C. longa, C. kwangsiensis*	Cai et al. (2018)
	Myrcene	C_10_H_16_	*C. wenyujin, C. longa*	Cai et al. (2018)
	Linalool	C_10_H_18_O	*C. wenyujin, C. longa,C. kwangsiensis*	Cai et al. (2018)

### 3.2 Curcuminoids

**FIGURE 2:**
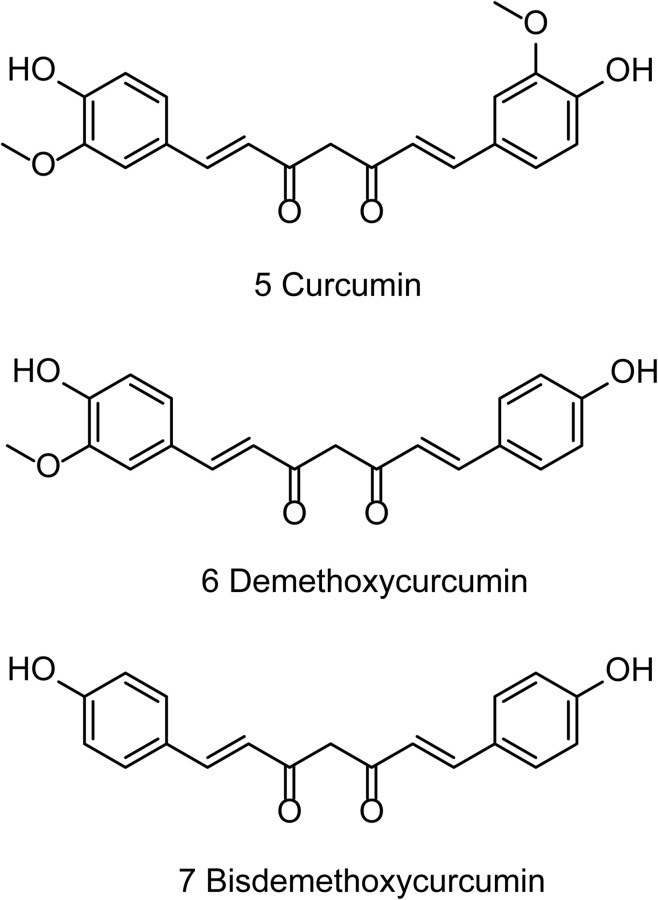
Chemical structures of representative curcuminoids in Curcumae Radix. (5) Curcumin, (6) Demethoxycurcumin, (7) Bisdemethoxycurcumin.

Curcuminoids are characterized by a diarylheptane backbone. Based on the substituents on the benzene rings, they can be classified into phenolic and non-phenolic types, mainly including curcumin, demethoxycurcumin, and bisdemethoxycurcumin (Figure 2). These compounds exhibit multiple pharmacological effects such as antitumor, anti-inflammatory, antioxidant, hepatoprotective, and neuroprotective activities. The content of curcuminoids varies significantly among different botanical origins of *Curcumae Radix*. *C*. *longa* contains the highest content of curcuminoids, followed by *C. wenyujin*, while *C. kwangsiensis* and *C. phaeocaulis* have relatively low contents (Lan et al., 2024).

### 3.3 Polysaccharides

Polysaccharides are one of the important active components of *Curcumae Radix*, exhibiting anti-fibrotic, blood glucose-regulating, and coagulation-modulating effects (Zhao et al., 2025). Their monosaccharide composition varies with the botanical origin: polysaccharides from *C. wenyujin* are mainly composed of arabinose, fructose, and glucose, whereas those from *C. kwangsiensis* primarily consist of glucose, galactose, and mannose (Guan et al., 2023). The polysaccharide content also differs considerably among origins, with *C. phaeocaulis* having the highest content (25.775%), followed by *C. kwangsiensis* (3.955%), and *C. longa* the lowest (2.695%) (Wang X. et al., 2012).

### 3.4 Other components

*Curcumae Radix* also contains various other types of chemical constituents: diterpenoids such as curcumenol A-F; triterpenoids such as drevinogenin II; alkaloids such as aurantiamide; sterols such as β-sitosterol and daucosterol (Su et al., 2025); phenolics and organic acids such as ferulic acid and vanillic acid; flavonoids such as luteolin (Yuan et al., 2016). In addition, it contains multiple trace elements including Fe, Mn, Zn, Cu, Co., Ni, Pb, and Cd, among which Fe, Co., and Zn are relatively abundant. These trace elements may participate in the regulation of enzyme activities and metabolic processes in the body (Wu et al., 2015).

### 3.5 Chemical differences among different botanical origins and candidate quality markers

In recent years, chemometrics has been widely used to distinguish the chemical differences among the four legally recognized botanical origins of *Curcumae Radix*. Using ultra performance liquid chromatography (UPLC) characteristic chromatograms combined with principal component analysis (PCA) and orthogonal partial least squares discriminant analysis (OPLS-DA), the four origins can be successfully differentiated, and the differential components contributing most to the classification have been identified, including turmerone, curcumenone, curdione, curcumenol, curcumol, dihydrocurcumin, demethoxycurcumin, and furanodienone (Wang A. Q. et al., 2022). Ultra high performance liquid chromatography coupled with quadrupole time of flight mass spectrometry (UHPLC-Q-TOF-MS) based metabolomics studies have also confirmed significant differences in the metabolite profiles among the four origins (Liu et al., 2016). Furthermore, near-infrared spectroscopy (NIR) combined with chemometrics enables rapid, non-destructive identification of botanical origins (Wang et al., 2021).

These studies provide a basis for the selection of quality markers (Q-markers) for *Curcumae Radix*. Curdione, germacrone, furanodiene, β-elemene, curcumin, and demethoxycurcumin are candidate Q-markers. Given the significant differences in component contents among different origins, it is necessary to establish chemical quality control standards based on characteristic components. Current research has mostly focused on volatile oils and curcuminoids, while the chemical characterization of polysaccharides, diterpenoids, and trace components remains insufficient. Moreover, the assignment and quantification of numerous trace-level sesquiterpenoids in the volatile oil still require improvement. Future efforts should strengthen the systematic analysis of each component group to provide a basis for the material basis research and precise quality control of *Curcumae Radix*.

Importantly, the chemical heterogeneity among the four origins implies potential differences in pharmacological profiles. However, most current studies have focused on chemical discrimination rather than pharmacological comparison. Therefore, whether origin-specific marker compounds can predict origin-specific therapeutic advantages remains largely unresolved. Establishing an origin-component-activity correlation is essential for transforming chemical quality evaluation into efficacy-oriented quality control.

## 4 Pharmacological effects

**FIGURE 3:**
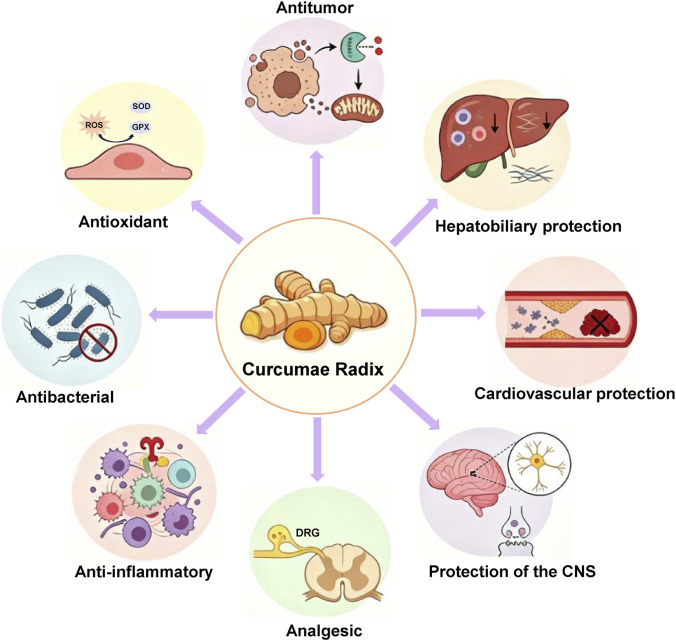
Overview of the main pharmacological activities of *Curcumae Radix*. The diagram summarizes the key pharmacological effects of *Curcumae Radix*, including antitumor, antioxidant, hepatobiliary protection, cardiovascular protection, anti-inflammatory, protection of the central nervous system (CNS), analgesic, and antibacterial activities. These effects are mediated by its major bioactive constituents (volatile oils and curcuminoids) through multi-target and multi-pathway mechanisms. Abbreviations: CNS, central nervous system; ROS, reactive oxygen species; SOD, superoxide dismutase; GPX, glutathione peroxidase; DRG, dorsal root ganglion.

In recent years, the pharmacological effects of *Curcumae Radix* and its extracts have been widely confirmed, exhibiting a broad-spectrum of biological activities in antitumor, hepatoprotection, anti-inflammation, analgesia, cardiovascular protection, and central nervous system (CNS) regulation. Its pharmacodynamically active substances are mainly volatile oils and curcuminoids, which exert effects through multi-target and multi-pathway synergistic regulation (Figure 3).

In this section, pharmacological evidence is organized according to both biological activities and evidence types. When available, evidence from purified compounds, volatile oil fractions, crude extracts, and whole medicinal materials is distinguished. It should be noted that purified compounds such as curcumin, β-elemene, curcumol, and germacrone cannot be directly equated with the pharmacological effects of whole *Curcumae Radix*. Therefore, conclusions regarding the efficacy of the medicinal material are drawn cautiously, especially when direct evidence from standardized extracts or origin-specific samples is lacking.

### 4.1 Antitumor activity

*Curcumae Radix* exhibits multi-target and multi-pathway antitumor activities. Its monomeric compounds, volatile oils, and crude extracts systematically intervene in tumor progression through inhibiting proliferation, inducing apoptosis, blocking metastasis and angiogenesis, reversing drug resistance, modulating the immune microenvironment, and regulating epigenetic modifications.

Among the active monomeric components, β-elemene is the most extensively studied and broad-spectrum antitumor compound in *Curcumae Radix*. It exerts antitumor effects by interfering with multiple steps of tumor development: in hepatocellular carcinoma and non-small cell lung cancer, it directly inhibits proliferation and induces apoptosis by downregulating long non-coding RNA HOX transcript antisense RNA (lncRNA HOTAIR) and activating the endoplasmic reticulum stress pathway, respectively (Wu et al., 2022; Liu et al., 2017). Furthermore, β-elemene exhibits dual functions of chemo/radiosensitization and microenvironment remodeling. By modulating pathways such as phosphatidylinositol three kinase/protein kinase B (PI3K/Akt) and nuclear factor-κB (NF-κB), it enhances the efficacy of cisplatin and 5-fluorouracil and ameliorates the immunosuppressive tumor microenvironment (Kun et al., 2020; Su et al., 2020; Wang Ma, 2024; Xie Q. et al., 2020). These effects collectively converge on the PI3K/Akt and NF-κB signaling hubs, suggesting that they constitute the molecular basis for the broad-spectrum activity of β-elemene.

Curcumin acts primarily at the epigenetic level by reversing aberrant DNA methylation to reactivate tumor suppressor genes (Fabianowska-Majewska et al., 2021). Simultaneously, it blocks tumor angiogenesis by downregulating vascular endothelial growth factor (VEGF) mRNA expression (He, 2006).

In addition, curcumol specifically induces apoptosis in p53-mutant triple-negative breast cancer by activating the p73 protein-p53 upregulated modulator of apoptosis/Bcl-2 antagonist (p73-PUMA/Bak) pathway (Huang et al., 2017). Curcumenol targets VEGF and its downstream signaling to inhibit angiogenesis and metastasis in hepatocellular carcinoma (Han et al., 2019). Diterpenoid C induces apoptosis in colon cancer cells by activating the p38 mitogen activated protein kinase (p38 MAPK) pathway and upregulating caspase-3 expression (Shen et al., 2014).

Regarding multi-component mixtures, the volatile oil of *C*. *wenyujin* acts as a multi-component synergistic system. It enhances the apoptotic effect on hepatocellular carcinoma cells by co-activating mitochondria and caspase-3 dependent apoptotic pathways (Xiao et al., 2008). Crude extracts of *Curcumae Radix* inhibit breast cancer cell migration and lung metastasis by downregulating C-C chemokine receptor 7 (CCR7), activator protein-1 (AP-1), and matrix metalloproteinase-9 (MMP-9) expression (Kaya et al., 2019). The n-butanol extract of *C. wenyujin* reverses multidrug resistance in gastric cancer cells by downregulating P-glycoprotein and GCS expression (Dai et al., 2014).

The antitumor effect of *Curcumae Radix* is characterized by synergistic regulation involving multiple components, multiple targets, and multiple pathways. Among these, elemene injection has been approved as a national Class II new antitumor drug, demonstrating clear clinical translational value (Tong et al., 2019; Wang S. X. et al., 2017; Zhai et al., 2018).

However, current research still has notable shortcomings: most evidence comes from *in vitro* or animal studies, with a lack of high-quality clinical data; synergistic or antagonistic interactions among components remain unclear; and systematic comparisons of antitumor activity among the four officially recognized botanical origins are lacking. It should be noted that although β-elemene and curcumol are mainly discussed in relation to *C. wenyujin*, most studies investigated isolated compounds rather than standardized extracts from different origins. Therefore, whether *C. wenyujin* exhibits superior antitumor activity among the four origins remains to be directly verified. Future efforts should strengthen clinically oriented translational research and integrate spectrum-effect relationships with metabolomics to elucidate the multi-component regulatory network, thereby providing a scientific basis for the precise antitumor application of *Curcumae Radix*.

### 4.2 Hepatoprotection

*Curcumae Radix* traditionally possesses the effect of breaking blood and moving qi, making it a commonly used medicinal herb for hepatobiliary diseases in traditional Chinese medicine (TCM) (Shi et al., 2020). Modern pharmacological studies have confirmed that its pharmacodynamic material basis includes monomeric components such as curcumin and crude extracts of *Curcumae Radix*.

Curcumin exerts hepatoprotective effects through anti-inflammatory and immunomodulatory mechanisms, significantly reducing the cytoplasmic translocation and expression of high mobility group box 1 (HMGB1), thereby alleviating acute liver injury induced by Propionibacterium acnes in mice (Gu et al., 2015). Curcumol alleviates ethanol induced hepatocyte senescence and improves the pathological status of alcoholic fatty liver disease (Qi et al., 2022).

The water decoction of *Curcumae Radix* antagonizes liver injury through dual pathways of anti-apoptosis and anti-oxidation, inhibiting excessive hepatocyte apoptosis while scavenging free radicals and reducing oxidative stress damage (Zhang et al., 2014). Extract of *C. wenyujin* significantly ameliorates liver fibrosis by blocking the transforming growth factor-β1 (TGF-β1)/Smad pro-fibrotic pathway, inhibiting hepatic stellate cell activation, and regulating extracellular matrix metabolism balance (Xie H. et al., 2020).

*Curcumae Radix* exerts hepatoprotective effects through mechanisms including anti-inflammation, anti-apoptosis, anti-oxidation, and anti-fibrosis, which are consistent with its traditional efficacy. However, current research mostly focuses on acute liver injury models, with a lack of clinical evidence for chronic liver diseases. Moreover, most studies involve curcumin, curcumol, or selected extracts, and few compare the four official origins. Considering the high curcuminoid content of *C. longa* and the sesquiterpenoid-rich profile of *C. wenyujin*, comparative studies in acute liver injury and liver fibrosis models are warranted.

### 4.3 Analgesic activity

*Curcumae Radix* possesses the effect of activating blood circulation to relieve pain and is clinically used for dysmenorrhea, hypochondriac distension and pain, and other pain symptoms caused by qi stagnation and blood stasis. Modern pharmacological studies have shown that its analgesic action involves the regulation of neurotransmitters, inhibition of inflammatory responses, and modulation of pain signaling pathways, with distinct mechanisms depending on the type of pain.

Curcumin alleviates chikungunya virus induced arthralgia and alcohol induced neuropathic pain (Sengupta et al., 2023). β-Elemene, when combined with morphine delivered via an intravenous analgesia pump in clinical practice, effectively reduces pain scores in patients with refractory cancer pain, with low adverse reactions (Cai et al., 2019).

The water decoction of *C*. *wenyujin* prolongs the writhing latency, reduces the number of writhing episodes, and increases the pain threshold in the acetic acid induced writhing model in mice, demonstrating clear analgesic activity (Zhang et al., 2020). Extracts of *Curcumae Radix* exhibit specific effects on different types of pain: in peripheral inflammatory pain, they alleviate formalin induced inflammatory pain by inhibiting NF-κB activation in the hippocampal CA1 region and reducing serum interleukin-1β (IL-1β) levels (Gong et al., 2016); in neuropathic pain, they increase the mechanical withdrawal threshold by downregulating spinal P2X purinoceptor 4 (P2X4) receptor expression and decreasing serum brain-derived neurotrophic factor (BDNF) levels (Hu et al., 2015; Li et al., 2015); in a dysmenorrhea model, they improve uterine smooth muscle spasm by modulating calcium signaling pathways and fatty acid metabolism (Qin et al., 2023a).

*Curcumae Radix* exerts safe and targeted analgesic effects based on its monomeric components (e.g., curcumin and β-elemene) and extracts, making it particularly suitable for long-term management of chronic pain and dysmenorrhea of the *qi* stagnation and blood stasis type. Existing analgesic studies mainly focus on *C. wenyujin*, *C. longa*, or processed products. Whether the four origins differ in their analgesic potency, especially in dysmenorrhea and inflammatory pain models, remains unclear. Future studies should conduct high-quality clinical randomized controlled trials for chronic pain and compare the differences in analgesic activity among different botanical origins.

### 4.4 Cardiovascular protection

*Curcumae Radix* has the effect of activating blood circulation and removing blood stasis. It is a commonly used medicinal herb for cardiovascular diseases in TCM, and its pharmacological effects are mainly reflected in antithrombotic activity, regulation of vascular function, and improvement of blood circulation (Hao et al., 2018).

Curdione enhances vasodilation and inhibits thrombosis (Wang X. H. et al., 2012); it also suppresses ferroptosis by regulating the kelch-like ECH-associated protein 1/thioredoxin 1/glutathione peroxidase 4 (Keap1/Trx1/GPX4) pathway, thereby alleviating isoproterenol-induced myocardial injury (Wang et al., 2023).

The volatile oil of *Curcumae Radix* downregulates the expression of myocardial hypertrophy-related genes such as Akt1, TNF, and MAPK3, inhibiting the progression of cardiac hypertrophy (Wang H. et al., 2025). *C*. *wenyujin* regulates lipid and amino acid metabolism to improve the pathological state in a rat model of acute blood stasis (Hao et al., 2018). The polysaccharide and non-polysaccharide components of *C. phaeocaulis* protect the vascular endothelium by inhibiting the sphingosine kinase 1 (SPHK1) pathway and improve hemorheology in septic rats (Zhou et al., 2026). The polysaccharides of *C*. *kwangsiensis* exert anticoagulant effects through the coagulation pathway (Yang et al., 2012), and its aqueous extract inhibits thrombosis by modulating the balance between thromboxane and prostacyclin (Su et al., 2019).

Studies have shown that all four botanical origins of *Curcumae Radix* possess significant antiplatelet aggregation activity without obvious differences among them, although *C. kwangsiensis* exhibits relatively stronger inhibitory effects (Jiang et al., 2015). Among the currently available pharmacological data, antiplatelet aggregation is one of the few endpoints for which different origins have been compared under similar conditions. This provides a useful example of how origin-specific efficacy may be investigated, but comparable evidence for other cardiovascular endpoints remains lacking.

In summary, the cardiovascular protective effects of *Curcumae Radix* cover multiple levels including blood, blood vessels, and myocardium, which are highly consistent with the needs for prevention and treatment of thrombotic diseases, coronary heart disease, and other cardiovascular disorders.

### 4.5 Protection of the central nervous system

*Curcumae Radix* exhibits multi-target and multi-pathway pharmacological activities in the regulation of CNS disorders, with particularly significant intervention potential in Alzheimer’s disease (AD) and depression.

#### 4.5.1 Anti-alzheimer’s disease

The volatile oil of *C*. *wenyujin* ameliorates neuronal pathological morphological abnormalities in the hippocampal region of AD model mice and improves learning and memory abilities (Qi et al., 2017a), with the mechanism involving activation of the PI3K/Akt signaling pathway and inhibition of tau protein hyperphosphorylation (Qi et al., 2017b). The ethyl acetate fraction of *Curcumae Radix* regulates the mitophagy pathway, thereby inhibiting neuronal pyroptosis, alleviating neuroinflammation, improving cognitive impairment, and reducing cerebral Aβ deposition (Qi et al., 2025). In summary, the anti-AD effect of *Curcumae Radix* targets three core pathological links: tau protein phosphorylation, mitochondrial functional homeostasis, and neuroinflammation.

#### 4.5.2 Antidepressant effects

Curcumin exerts antidepressant effects at multiple levels: at the neurotransmitter level, it corrects imbalances of 5-hydroxytryptamine (5-HT), dopamine (DA) and inhibits glutamate excitotoxicity (Lin G. B. et al., 2011; Xia et al., 2007); at the neurotrophic level, it upregulates BDNF expression to promote neuronal repair (Kumar, 2008); at the neuroinflammatory level, it exerts anti-inflammatory effects by inhibiting the production of inflammatory mediators and NF-κB activation (Arora et al., 2011; Hong et al., 2004). Curzerene improves depression-like behavior and cognitive impairment by inhibiting HMGB1-related inflammatory pathways and restoring gut microbiota homeostasis (Huang et al., 2025). The herbal pair of *Curcumae Radix* and *Acori Tatarinowii Rhizoma* effectively alleviates depression-like behavior by regulating microglial M1/M2 polarization and inhibiting neuronal apoptosis. Its synergistic regulation of the brain-gut-immune network represents an important advantage over single-target drugs (Lin et al., 2026).

The antidepressant effect of *Curcumae Radix* covers multiple levels including neural, inflammatory, and gut-brain axis pathways, which is consistent with the complex pathogenesis of depression and suggests its potential for further investigation as a natural antidepressant candidate. However, the onset time, optimal dosage, and long-term safety still require clinical confirmation.

### 4.6 Anti-inflammatory

The anti-inflammatory effect of *Curcumae Radix* is characterized by multi-component, multi-target, and multi-pathway holistic regulation. The core mechanism centers on the NF-κB signaling pathway, which simultaneously regulates upstream toll-like receptor 4 (TLR4) signaling and downstream inflammatory factor networks, forming a multi-level anti-inflammatory effect.

Components such as diterpenoid C from *C*. *wenyujin*, β-elemene, and the sesquiterpenes and diterpenes from *C*. *kwangsiensis* all inhibit the release of pro-inflammatory cytokines and balance the inflammatory microenvironment by modulating the NF-κB and related signaling pathways (Huang et al., 2013; Xie Q. et al., 2020; Yuan et al., 2020). Curcumin reduces IL-1β levels in chronic inflammation models, suppresses endoplasmic reticulum stress and the NF-κB pathway, and exerts protective effects against renal inflammation (Zhong et al., 2011).

The volatile oil of *Curcumae Radix* blocks the TLR4/NF-κB pathway, inhibits macrophage M1 polarization and T cell differentiation, and improves lung function in a pulmonary sarcoidosis model (Mao et al., 2025). The essential oil of *C. wenyujin* exerts anti-inflammatory effects against acute gouty arthritis by regulating this pathway and alleviating oxidative stress (Meng et al., 2025).

The extract of *C*. *kwangsiensis* significantly inhibits xylene induced ear edema, cotton pellet induced granuloma proliferation, and increased capillary permeability, demonstrating broad-spectrum anti-inflammatory activity in acute inflammation models (Lin T. Y. et al., 2011). The polysaccharide and non-polysaccharide components of *Curcumae Radix* reduce inflammatory cytokine levels, alleviate multi-organ damage, and delay the progression of sepsis by inhibiting the sphingosine kinase 1 (SPHK1) signaling pathway (Zhou et al., 2026).

Current evidence suggests that the anti-inflammatory effects of *Curcumae Radix* are closely associated with NF-κB-related pathways and involve a broad range of inflammatory models. It serves as the common basis for multiple pharmacological effects of *Curcumae Radix*, including antitumor, hepatoprotective, analgesic, and neuroprotective activities, and is particularly suitable for the intervention and treatment of chronic inflammation related diseases.

### 4.7 Other effects

The volatile oils and curcuminoids in *Curcumae Radix* are the main material basis for its antibacterial activity. They exhibit significant inhibitory effects against a variety of Gram-positive and Gram-negative pathogenic bacteria, including *Staphylococcus aureus*, *Pseudomonas aeruginosa*, *Shigella dysenteriae*, *Shigella* species, and *Bacillus subtilis* (Gao et al., 2016; Huang et al., 2016). This broad-spectrum antibacterial property provides an experimental basis for the application of *Curcumae Radix* in infectious diseases.

The ethanol extract of *Curcumae Radix* exhibits a protective effect against hydrogen peroxide-induced oxidative stress injury in human umbilical vein endothelial cells (HUVECs). The underlying mechanism is associated with enhancing antioxidant enzyme activities, alleviating oxidative damage, promoting vasodilation, inhibiting inflammatory responses and cell apoptosis, and regulating the expression of apoptosis-related genes (Tang et al., 2019). This suggests that the antioxidant activity of *Curcumae Radix* serves as an important basis for its vascular endothelial protection, anti-inflammatory, and anti-apoptotic effects.

In summary, the pharmacological effects of *Curcumae Radix* cover multiple areas including antitumor, hepatoprotection, analgesia, cardiovascular protection, neuroprotection, and anti-inflammation. Its pharmacodynamic material basis primarily consists of volatile oils (especially sesquiterpenoids) and curcuminoids. These active components do not act in isolation but form a synergistic regulatory network through interwoven multi-target and multi-pathway mechanisms. Notably, there are close intrinsic connections among the different pharmacological effects. For instance, anti-inflammation serves as a common hub for many of the therapeutic actions of *Curcumae Radix*. On the one hand, inhibition of inflammatory pathways directly mediates its anti-inflammatory effect, while also participating in antitumor activity (e.g., reversing the immunosuppressive microenvironment), analgesia (e.g., reducing the release of inflammatory factors), and hepatoprotection (e.g., alleviating inflammatory liver injury) (Gong et al., 2016; Gu et al., 2015; Xie H. et al., 2020). On the other hand, the analgesic and antitumor effects of *Curcumae Radix* form a functional synergy in the treatment of cancer pain. Elemene injection not only inhibits tumor growth but also alleviates refractory cancer pain (Cai et al., 2019; Qureshi et al., 2019), demonstrating the advantage of addressing both the root cause and symptoms.

Despite the broad-spectrum pharmacological activities of *Curcumae Radix*, the current research still has the following limitations. The level of evidence is relatively low, mostly limited to *in vitro* or animal models, with a lack of high-quality clinical translational studies. Most studies focus on single components (e.g., β-elemene, curcumin) or crude extracts, while reports on whether synergistic or antagonistic effects exist among multiple sesquiterpenoids in the volatile oil, or between sesquiterpenoids and curcuminoids, are extremely scarce. The chemical profiles of different botanical origins differ significantly, yet comparative pharmacological studies are seriously insufficient. Future research should strengthen comparative pharmacology based on clinical efficacy, clarify the dominant indications of different botanical origins, systematically analyze the synergistic/antagonistic patterns among multiple components, and integrate spectrum-effect relationships with metabolomics, thereby providing a scientific basis for the precise application of *Curcumae Radix*.

## 5 Clinical applications

According to the Pharmacopoeia, *Curcumae Radix* is indicated for stabbing pain in the chest and hypochondria, chest impediment with heart pain, amenorrhea and dysmenorrhea, breast distension and pain, unconsciousness in febrile diseases, epilepsy and mania, blood heat-related epistaxis and hematemesis, as well as jaundice with dark urine (Committee, 2025). In clinical practice, following the principle of syndrome differentiation and treatment in TCM, it achieves synergistic effects through compound formulations, with broad application in traditional and contemporary clinical practice. Its application in traditional formulas encompasses classical famous prescriptions, commonly used herb pairs, and various clinical compatibility patterns, thereby demonstrating unique clinical value in hepatobiliary, cardiovascular, neuropsychiatric, and dermatological diseases.

*Curcumae Radix* follows the principles of syndrome differentiation and treatment in TCM, achieving synergistic effects through compatibility with other herbs. It has been widely used in multiple systemic diseases according to traditional experience and modern clinical observations; however, the overall level of clinical evidence remains limited.

The classic famous formula Changpu Yujin Tang, composed of *Acori Tatarinowii Rhizoma*, *Curcumae Radix*, *Gardeniae Fructus*, *fresh Lophatheri Herba*, and *Moutan Cortex* (Tian et al., 2025), is commonly used in the integrative treatment of epidemic encephalitis B, multiple tic disorder, sleep disorders, and viral encephalitis (Chen et al., 2023; Gao et al., 2021; Jing, 2026; Li H. L. et al., 2022; Wang et al., 2011).

When *Curcumae Radix* is used in combination with Angong Niuhuang Wan, it serves as a minister herb, synergizing with the sovereign herbs *Bovis Calculus* and *Moschus* to enhance the effects of clearing heat, detoxifying, opening the orifices, and awakening the spirit. This combination helps improve impaired consciousness in patients with stroke, encephalitis, and critically ill patients during recovery (General Office of National Health Commission of the People’s Republic of China, 2020).

The combination of *Curcumae Radix* with *Bupleuri Radix* is a common pairing for the treatment of fatty liver disease. It enhances the effects of soothing the liver, activating blood, resolving stasis, and unblocking collaterals, thereby improving local blood circulation in the liver (Wu et al., 2020). In the integrative treatment of choledocholithiasis, Chaihu Yujin Paishi Decoction combined with epidural anesthesia shows significant efficacy. It promotes the discharge of gallstones by regulating biliary motility, reducing inflammatory responses, and alleviating smooth muscle spasm (Wang, 2018).

In addition to the above classic famous formulas and commonly used herb pairs, *Curcumae Radix* has various other compatibility applications in traditional compound prescriptions. These involve digestive system diseases (e.g., superficial gastritis, chronic atrophic gastritis, intractable hiccup, deficient-cold stomach pain, neurogenic vomiting) (Du, 2010; Guo, 2013; Wang et al., 2026), neuropsychiatric disorders (e.g., post-stroke depression, coronary heart disease with comorbid depression and anxiety) (Chen J. Y. et al., 2025; Fan and Chang., 2010), skin diseases (psoriasis vulgaris) (Chen et al., 2017; Zhang et al., 2016), as well as infectious diseases (early-stage COVID-19, gynecological infections), breast hyperplasia, postherpetic neuralgia, angina pectoris of coronary heart disease, and eruption in damp-warm disease, among other conditions (Hu et al., 2016; Huang et al., 2016; Lin et al., 2020; Song, 2010; Tian and Zhou, 2010; Wang J. Z. et al., 2017).

However, the clinical evidence supporting these combinations is mostly limited to small-sample case series, expert opinions, or classic medical literature. There is a lack of validation through randomized controlled trials or large-scale prospective studies. To avoid omitting information that may have certain clinical reference value, these compatibility applications are summarized in Table 3.

**TABLE 3:** Other compatibility applications of *Curcumae Radix* in compound formulas.

Compatibility formula	Disease area	Main use	Ref
Curcumae Radix-based formula	Acute viral hepatitis	Alleviate fatigue, poor appetite, nausea, abdominal distension; reduce transaminase levels	Zhang (1992)
Modified Yujin Powder	Superficial gastritis	Relieve stomach pain, bloating, belching	Du (2010)
Erchen Decoction plus Changpu Yujin Decoction	Chronic atrophic gastritis	Total effective rate 92.6%	Zhang et al. (2026)
Modified Dingxiang Yujin Powder	Intractable hiccup	Definite efficacy	Zhou and Deng (2008)
Dingxiang plus Curcumae Radix (modified according to symptoms)	Deficient-cold stomach pain	Improve symptoms	Guo (2013)
Yujin Dingxiang Heye Decoction	Neurogenic vomiting	Commonly used formula	Wang et al. (2021)
Curcumae Radix plus Acori Tatarinowii Rhizoma, Arisaema cum Bile, Polygalae Radix (combined with fluoxetine)	Post-stroke depression	Alleviate depression, improve limb function	Fan and Chang (2010)
Curcumae Radix plus Codonopsis Radix, Poria, Glycyrrhizae Radix	Coronary heart disease with comorbid depression and anxiety	Relieve stagnation, move qi, support yang	Chen et al. (2025)
Yujin Yinxie Tablets plus Compound Amino-polypeptide Tablets	Psoriasis vulgaris	Total effective rate >95%	Zhang (2016)
Kangbingdu Oral Liquid (containing Curcumae Radix)	Early COVID-19	Alleviate fever, fatigue, cough	Lin et al. (2020)
Baofukang Suppository (volatile oil of C. wenyujin)	Gynecological infection	Mycotic vaginitis, cervical erosion	Huang et al. (2016)
Curcumae Radix plus Bupleuri Radix	Breast hyperplasia	Regulate qi flow, activate blood circulation, relieve stasis	Wang S. X. et al. (2017)
Curcumae Radix plus Caryophylli Flos, Bupleuri Radix	Postherpetic neuralgia	Soothe the liver, regulate qi, activate blood circulation, unblock collaterals, and relieve pain	Song (2010)
Curcumae Radix plus Caryophylli Flos	Angina pectoris	Warm and unblock the heart vessels	Tian et al. (2010)
Curcumae Radix plus Armeniacae Semen	Damp-warm disease with skin eruptions	Skin rashes, nighttime insomnia	Hu et al. (2016)

Despite the widespread clinical use of compound prescriptions containing *Curcumae Radix*, the level of clinical evidence remains low. Furthermore, the common practice of indiscriminate use of different botanical origins compromises the consistency and reproducibility of therapeutic efficacy. In the future, high-quality clinical studies should be conducted in the dominant therapeutic areas of *Curcumae Radix*, and standardized randomized controlled trials should be performed on classic compound formulas to clarify the indication positioning of different botanical origins, thereby promoting evidence-based and precise clinical application of *Curcumae Radix*.

## 6 Toxicology

As a TCM, *Curcumae Radix* has a medicinal history of over a thousand years. Modern toxicological experiments and clinical studies have confirmed its favorable overall safety profile and low acute toxicity, allowing safe use at conventional doses. Potential risks occur only at excessively high doses or after long-term administration, or combination with specific drugs.

### 6.1 Toxicity

The acute toxicity of *Curcumae Radix* is extremely low, with a median lethal dose (LD50) far exceeding the clinically equivalent dose. Acute toxicity experiments in mice via intragastric administration showed that the LD50 values of the four botanical origins of *Curcumae Radix* were all significantly higher than the maximum clinically recommended dose. Among them, *C*. *wenyujin*, which exhibited the highest toxicity, had an LD50 of 80.98 g/kg, equivalent to 485.9 times the maximum daily clinical dose for adults. No obvious signs of toxicity were observed after a single administration, and no significant pathological damage was found in the major organs (Song et al., 2011).

Long-term safety of *Curcumae Radix* is favorable, with no significant cumulative toxicity. Rats continuously administered *Curcumae Radix* extract (21.86 g/kg bw) by gavage showed no signs of toxicity or mortality. At a dose of 1.875 mL/kg bw, no abnormalities were observed in rat behavior, body weight, routine blood parameters, blood biochemical indices, or histopathological examination. This dose can be considered the no observed adverse effect level (Zhu et al., 2018). In addition, studies have found that the ethanol extract of *Curcuma Radix* aromatica exhibits no obvious genotoxicity, indicating good oral safety (Mo et al., 2015).

### 6.2 Compatibility contraindications

*Curcumae Radix* and *Caryophylli Flos* are included in the “Nineteen Incompatibilities”, a traditional theory that considers them mutually antagonistic. However, studies have shown that the combination of *Curcumae Radix* and *Caryophylli Flos* has high practical value, but its safety mainly depends on the dosage ratio. Eugenol, a component of *Caryophylli Flos*, can cause gastric mucosal congestion, promote gastric juice secretion, and enhance gastrointestinal motility (Wu et al., 2025). If the dosage of *Caryophylli Flos* is too high and used together with *Curcumae Radix*, it may cause adverse reactions such as vomiting and gastrointestinal bleeding (Wang et al., 2021). Whether this combination constitutes an absolute compatibility contraindication as described in the traditional “Nineteen Incompatibilities” remains unconfirmed due to the lack of systematic and standardized clinical and experimental studies, and further verification is required.

## 7 Processing

Processing is a unique pharmaceutical technology in TCM. Most TCM and raw materials for herbal preparations require standardized processing before clinical use. During the processing procedure, various biochemical and chemical changes occur, altering the pharmacodynamic material basis to moderate medicinal properties, reduce toxicity, enhance therapeutic efficacy, and increase synergistic effects. Processing also serves as an important foundation for the modernization, industrialization, and internationalization of TCM.

### 7.1 Traditional processing methods

The processing of *Curcumae Radix* has a long history, first recorded in the Tang Dynasty. Through continuous development and refinement during the Song, Yuan, Ming, and Qing dynasties, a technical system gradually emerged. This system is based on cleaning and cutting, with vinegar-processing and wine-processing as core methods, supplemented by baking, stir-frying, simmering, burning, and various adjuvant-based processing techniques. The purpose of processing has evolved from the initial “removing impurities and facilitating pulverization” to guiding the medicinal action to specific channels, enhancing efficacy while reducing disadvantages, and directionally regulating medicinal properties (Chen et al., 2018). Among these, cleaning and cutting aim to remove impurities and promote the dissolution of components (Jin, 1982; Wang, 1958). Heating-based methods such as baking, stir-frying, simmering, and burning can alter medicinal properties and facilitate storage (Wang, 2012; Zhang, 2005; Zhu, 1959). Vinegar-processing directs the action to the liver and enhances the effects of soothing the liver, moving qi, and relieving pain (Fu, 2007; Li, 2015; Miao, 2013). Wine-processing enhances the effects of activating blood, resolving stasis, moving qi, and unblocking collaterals (Miao, 2013). Various adjuvant-based methods, such as processing with *Gleditsiae Sinensis Fructus* water, *Glycyrrhizae Radix* water, or alum water, reflect the concept of “syndrome-based processing and medication according to needs” (Liu, 2007; Zhou et al., 2025; Zhu, 2005).

### 7.2 Modern processing technology research

Modern research has promoted the development of *Curcumae Radix* processing toward parameterization and standardization. All editions of the Pharmacopoeia have stipulated the basic requirements of “washing, moistening thoroughly, cutting into thin slices, and drying in the sun”. Meanwhile, the processing standards of various regions specify that vinegar-processed and wine-processed *Curcumae Radix* are the commonly used decoction pieces in clinical practice.

#### 7.2.1 Cleaning and cutting

Modern technology employs rapid rinsing with running water to protect the integrity of the medicinal material epidermis (Liu et al., 2015). To address the low efficiency and component loss associated with traditional moistening and cutting methods, direct crushing using a sieve-plate-removed pulverizer can be adopted (Feng, 1988). Optimized pressurized soaking and softening processes can promote component dissolution and uniform softening. Drying should be carried out at room temperature or low temperature (≤40 °C) to avoid the loss of volatile oils (Huang et al., 2005).

#### 7.2.2 Vinegar-processing technology

Orthogonal tests and response surface methodology have been used to optimize the vinegar-processing technology, using the appearance of the decoction pieces, curcumin, and germacrone as evaluation indicators. The optimal procedure involves adding an appropriate amount of vinegar to clean *Curcumae Radix* slices, moistening, and then stir-frying, with slight variations in parameters among different botanical origins (Gu et al., 2017; Quan, 2019; Shi et al., 2011).

#### 7.2.3 Wine-processing technology

Orthogonal tests and response surface methodology have also been employed to optimize wine-processing technology, using germacrone, furanodienone, and curcumin as indicators. Differences in wine-processing parameters exist among different botanical origins. These studies provide data support for the standardization of wine-processed *Curcumae Radix* technology (Quan et al., 2019; Zhao et al., 2018; Zhao, 2018).

### 7.3 Effects of processing on chemical constituents

During vinegar-processing, the contents of curdione and germacrone in *C*. *wenyujin* decrease. Meanwhile, curdione undergoes tautomerism with the migration of hydrogen atoms and σ-bonds, converting into curcumol, resulting in a relative increase in its content (Zhou C. X. et al., 2017). Vinegar-processing can increase the dissolution of curcumin and promote molecular rearrangement and oxidation reactions, generating new components such as β-elemene (Wang M. et al., 2022). After vinegar-processing, the contents of active components such as curzerene, germacrene D, and β-elemene increase, while those of acetone and camphene decrease. Additionally, limonene is oxidized to produce limonene oxide, which is unique to vinegar-processed products (Qin et al., 2023b).

Different processing methods significantly affect the content of germacrone. The highest content is found in raw products mixed with vinegar, followed by raw products and stir-fried products mixed with vinegar, which are comparable. Stir-fried products alone have a lower content, and vinegar-processed products have the lowest. The addition of vinegar alone promotes dissolution, while heating leads to volatilization. Vinegar-processed products suffer the greatest loss. Stir-fried products mixed with vinegar have similar germacrone content to raw products, suggesting a balance between the dissolution-promoting effect of vinegar and the volatilization caused by heating (Shi, 2013).

Heating alone reduces the curcumin content in *C*. *wenyujin*, as curcumin is prone to decomposition or transformation under high temperature. In contrast, the addition of vinegar alone increases its content, which is related to the acidic environment of vinegar promoting curcumin dissolution. The effects of heating followed by vinegar addition and vinegar addition followed by heating on curcumin are consistent, both resulting in higher curcumin content than stir-fried products alone. This indicates that vinegar can partially offset the loss of curcumin caused by heating (Shi et al., 2013).

A new component, 5-hydroxymethylfurfural (5-HMF), which is not detected in raw products, is generated in vinegar-processed *Curcumae Radix* products. Its formation is related to the Maillard reaction: polysaccharides and reducing sugars undergo heating-induced dehydration and degradation to generate 5-HMF, and the acidic environment of vinegar accelerates this reaction (Gong et al., 2019). However, the specific pharmacological contribution of 5-HMF in vinegar-processed *Curcumae Radix* remains unclear and requires further experimental validation.

These processing-induced chemical changes provide a chemical basis for altered pharmacological activity; however, changes in content alone cannot fully explain efficacy enhancement unless they are further linked to bioavailability, tissue distribution, and pharmacodynamic outcomes.

### 7.4 Effects of processing on pharmacological activity

Processing can significantly modulate the pharmacological activity of *Curcumae Radix*, with particularly prominent effects in anti-liver fibrosis, analgesia, promoting blood circulation and removing blood stasis, and myocardial protection.

In terms of anti-liver fibrosis, vinegar-processed *Curcumae Radix* exhibits more significant effects compared with the raw product (Xie Q. et al., 2020). In addition, carbonized *Curcumae Radix* also exerts anti-liver fibrosis effects through anti-inflammatory, antioxidant, and liver injury repair pathways (Zhao et al., 2023). Vinegar-processed *Curcumae Radix* is commonly used in combination with vinegar-processed *Bupleuri Radix* and wine-processed *Paeoniae Radix Alba* for the treatment of liver and gallbladder qi stagnation syndrome (Zhang, 2017).

Regarding analgesic effects, processing markedly enhances the therapeutic action of *Curcumae Radix* in dysmenorrhea. Vinegar-stir-fried *C. longa* shows significantly enhanced analgesic effects. It reduces the writhing response in dysmenorrhea rats with liver qi stagnation, improves the pathological state of uterine tissue, and decreases the expression of cyclooxygenase-2 (COX-2) and oxytocin receptor (OTR) (Wu et al., 2024). The mechanism is closely related to the increased blood components such as curcumol and protocurcumenol after vinegar-processing, which subsequently regulate the estrogen signaling pathway (Peng et al., 2023). After vinegar-stir-frying or wine-stir-frying, the content of curcuminoids in *C*. *longa* increases, which can alleviate pain by regulating 5-HT release, increasing β-EP content, and inhibiting c-fos expression (Chen et al., 2020). Further studies have shown that vinegar-processed *Curcumae Radix* has better efficacy than the raw product in rats with primary dysmenorrhea. It improves uterine morphology and alleviates glandular hypertrophy, myometrial hyperplasia, and neutrophil infiltration. The mechanism involves the regulation of metabolic pathways such as pyrimidine, pyruvate, phenylalanine, and tyrosine (Su et al., 2022). However, the specific molecular mechanism by which vinegar-processing enhances analgesic effects still requires further elucidation.

After wine-processing, the effect of *Curcumae Radix* on promoting blood circulation and removing blood stasis is enhanced. This may be achieved by enhancing the regulation of the nitric oxide synthase (NOS)/NO system and the arachidonic acid cyclooxygenase metabolic pathway, thereby inhibiting platelet aggregation, dilating blood vessels, and protecting the vascular endothelium (Peng et al., 2022). In addition, carbon dots derived from calcined *Curcumae Radix* treat myocardial ischemia by reducing excessive oxidative stress in myocardial tissue and inhibiting cardiomyocyte apoptosis (Dong et al., 2024).

Currently, research on the correlation between processing-induced chemical changes and pharmacological activity modulation of *Curcumae Radix* remains weak. Although processing (e.g., vinegar- or wine-processing) is known to alter the contents of curcumin, germacrone, curdione, curcumol, β-elemene, and other volatile/non-volatile constituents, the direct causal relationship between these chemical changes and enhanced efficacy (e.g., anti-fibrosis, analgesia, blood circulation promotion) has not been systematically established. Moreover, the modern pharmacological interpretation of traditional processing theories—such as vinegar-processing “guiding the drug to the liver” or wine-processing “enhancing blood-activating effects” is still lacking. Specifically, whether vinegar-processing increases hepatic exposure or promotes liver-targeted distribution of active constituents, and whether wine-processing improves their bioavailability or vascular distribution, remain unknown. Future studies should integrate pharmacokinetics, tissue distribution, metabolomics, and pharmacodynamics to elucidate the causal chain of “chemical transformation, *in vivo* exposure, target distribution, pharmacological efficacy”, thereby providing a modern scientific basis for processing-induced efficacy enhancement.

## 8 Conclusions and future perspectives

*Curcumae Radix* is a Chinese medicinal herb that promotes blood circulation and moves qi, with effects including pain relief, depression resolution, blood cooling, and jaundice reduction. It is widely used in hepatobiliary, oncological, cardiovascular and cerebrovascular, neurological, and dermatological diseases. This article systematically reviews the chemical constituents, pharmacological effects, clinical applications, and processing research progress of *Curcumae Radix*. The herb mainly contains volatile oils (primarily sesquiterpenoids) and curcuminoids, with over 250 compounds having been identified. Pharmacological studies have shown that it possesses antitumor, hepatoprotective, anti-inflammatory, analgesic, cardiovascular protective, and neuroregulatory effects (Niu et al., 2024). *Curcumae Radix* is commonly combined with *Bupleuri Radix*, *Acori Tatarinowii Rhizoma*, and other herbs to synergistically treat hepatobiliary, neurological, and digestive system diseases (Chen et al., 2023; Li, 2022). The processing of *Curcumae Radix* has a long history, with techniques such as cleaning, cutting, vinegar-processing, and wine-processing having been developed (Chen, 2018). After processing, especially vinegar-processing, *Curcumae Radix* shows enhanced pharmacological effects in experimental models of liver fibrosis and dysmenorrhea-related pain, although clinical validation remains insufficient.

Despite significant progress in research, the following key issues still constrain the in-depth development and precise clinical application of *Curcumae Radix*. First, the translational pathway from chemical constituents to clinical efficacy remains unclear. The pharmacological mechanisms of most constituents are still at the stage of cellular or animal models. Moreover, the quantitative spectrum-effect relationship between processing-induced chemical changes and corresponding alterations in pharmacological activity has not yet been elucidated. Second, the relationships among botanical origins, processed products, and clinical indications have not been clarified. Although chemical composition and bioactivity vary significantly among different origins, they are often used interchangeably in clinical practice. The clinical selection among raw, vinegar-processed, and wine-processed *Curcumae Radix* lacks evidence-based guidelines. Third, clinical evidence in the distinctive advantageous areas is insufficient. Most supportive evidence comes from small-sample trials, and there is a lack of high-level studies such as multicenter, randomized double-blind trials.

Future research on *Curcumae Radix* should be fundamentally guided by TCM theory and oriented toward precise clinical application, focusing on key areas for systematic investigation. Specifically, the following four aspects should be pursued. First, an integrated system linking botanical origin, processing method, chemical markers, pharmacodynamics/indications should be established. Through systematic comparative pharmacology and metabolomics studies, the dominant disease conditions for different botanical origins of *Curcumae Radix* and their processed products in core therapeutic areas such as analgesia, anti-liver fibrosis, and antitumor activity should be clarified. Scientifically precise clinical selection guidelines should be developed to provide theoretical support for rational clinical medication. Second, translational research in characteristic advantageous areas should be deepened. High-quality clinical studies on elemene should be conducted focusing on key clinical conditions such as cancer pain management and liver fibrosis. Using evidence-based medicine approaches, clinical application guidelines for different processed products of *Curcumae Radix* in common diseases such as dysmenorrhea, hypochondriac pain, chest impediment, and depression should be further refined, thereby improving the standardization and efficacy of clinical application. Third, a multi-dimensional integrated quality control model should be established. Using modern analytical techniques such as fingerprint profiling and metabolomics, specific quality markers closely related to the core pharmacodynamic effects of *Curcumae Radix* should be screened. Multi-dimensional and refined quality control standards should be developed to ensure the uniformity, stability, and controllability of *Curcumae Radix* crude drugs and products. Fourth, active efforts should be made to promote the development of new drugs based on *Curcumae Radix*. *Curcumae Radix* possesses dual antitumor and analgesic effects. Existing studies have confirmed its potential therapeutic role in cancer pain (Cai et al., 2019; Qureshi et al., 2019). Current cancer pain management still faces many limitations due to issues such as drug addiction (Wang W. L. et al., 2025). As a TCM with low toxicity and high safety, *Curcumae Radix* provides a solid foundation for new drug development. Therefore, future research should focus on the direction of cancer pain treatment by conducting innovative drug development based on the active ingredients or fractions of *Curcumae Radix*, thereby providing safe and effective new options for clinical practice. In addition, depression is also a major clinical challenge that urgently needs to be addressed. *Curcumae Radix* has shown preliminary potential in antidepressant effects, and its potential for antidepressant new drug development deserves further exploration.

In summary, through in-depth exploration of active components, elucidation of pharmacological mechanisms, standardized guidance for clinical application, thorough interpretation of processing principles, and systematic improvement of quality standards, *Curcumae Radix*, as a TCM, is expected to achieve greater development in the process of modernization, thereby providing more precise, safer, and more effective therapeutic options for clinical practice.
